# Evaluation of the correlation of vasculogenic mimicry, Notch4, DLL4, and KAI1/CD82 in the prediction of metastasis and prognosis in non-small cell lung cancer

**DOI:** 10.1097/MD.0000000000013817

**Published:** 2018-12-28

**Authors:** Yichao Wang, Ruixue Yang, Xiaolin Wang, Hongfei Ci, Lei Zhou, Bo Zhu, Shiwu Wu, Danna Wang

**Affiliations:** a Department of Pathology, The First Affiliated Hospital of Bengbu Medical College; b Department of Pathology, Bengbu Medical College, Bengbu, Anhui Province, China.

**Keywords:** DLL4, KAI1/CD82, Notch4, NSCLC, prognosis, VM

## Abstract

Vasculogenic mimicry (VM) is a new blood supply style in tumors and has long been treated as a useful factor in malignant tumor metastasis and prognosis. Notch4 (a marker of Notch signaling pathway receptors), DLL4 (a marker of Notch signaling pathway ligands) and KAI1/CD82 (a suppressor gene of tumor metastasis) are all effective predictive factors for tumor metastasis. In this study, we analyzed correlations among VM, Notch4, DLL4, and KAI1/CD82 in non-small cell lung cancer (NSCLC), and their respective associations with patients’ clinicopathological parameters and survival rate in NSCLC.

Positive rates of VM, Notch4, DLL4, and KAI1/CD82 in 189 whole NSCLC specimens were detected by histochemical and immunohistochemical staining. Moreover, patients’ clinicopathological information was also collected.

Positive rates of VM, Notch4, and DLL4 were significantly higher, and levels of KAI1/CD82 were significantly lower in NSCLC than in normal lung tissues. Positive rates of VM, Notch4, and DLL4 were positively associated with tumor size, lymph node metastasis (LNM), distant metastasis (DM) and tumor-node-metastasis (TNM) stage, and inversely with patients, overall survival (OS) time and positive rate of DLL4 were positively associated with tumor grade. Levels of KAI1/CD82 were negatively associated with tumor size, LNM, DM, and TNM stage. The KAI1/CD82+ subgroup had significantly longer OS time than did the KAI1/CD82- subgroup. In multivariate analysis, high VM, Notch4, DLL4 levels, tumor size, LNM, DM, TNM stage, and low KAI1/CD82 levels were potential to be independent prognostic factors for overall survival time (OST) in NSCLC patients.

VM and the expression of Notch4, DLL4, and KAI1/CD82 represent promising markers for tumor metastasis and prognosis, and maybe potential therapeutic targets for NSCLC.

## 1 Introduction

In recent years, along with changes in environment and lifestyle, the incidence of lung cancer has increased to become the most commonly diagnosed cancer worldwide.^[1]^ Approximately 85% of lung cancer cases are non-small cell lung cancer (NSCLC), NSCLC includes 2 major histological types, adenocarcinoma (Ade) and squamous cell carcinoma (SCC).^[2]^ In spite of the progress in early diagnosis, surgical radical treatment, and molecular targeted therapies, 5-year survival rate for lung cancer patients remains less than 20%.^[3]^ Tumor recurrence and metastasis may be the main reasons for poor prognosis,^[4]^ therefore, it is still needed to further investigate potential prognostic markers and novel therapeutic targets for NSCLC.

Despite the use of conventional anti-angiogenic therapy, the patient's survival time did not significantly improve.^[5]^ Vasculogenic mimicry (VM) in tumor may represent one of the resistance mechanisms which have poor anti-angiogenic effects in clinical practice.^[6]^ The VM was first identified in melanoma in 1999.^[7]^ When endothelium-dependent vascular growth cannot sufficient to support rapidly growth of tumor tissue, some cancer cells can mimic endothelial cells, and form VM structure.^[7,8]^ VM is mainly composed of 3 structures: the lumen-like structure, stem-like cancer cells, and remodeling of the extracellular matrix (ECM), which can directly connect with the host microcirculation system.^[8,9]^ A series of studies have shown that cancer patients associated with VM structures are prone to tumor metastasis and have poor prognosis.^[8–10]^

Notch signaling pathway is an important form of cell-to-cell communication, which plays an important role in regulating stem cell proliferation, differentiation, and apoptosis during embryonic development.^[11]^ In mammals, there are 4 Notch receptors (Notch 1-4) and 5 ligands (Delta-like 1, 3, 4, and Jagged 1, 2).^[11,12]^ Deregulated expression of Notch receptors and their ligands has been observed in several human malignancies including NSCLC.^[11–14]^ Notch4 is specifically distributed in the vascular endothelium, which can regulate the formation and maturation of the vascular network structure in the late stage of blood vessel development, and inhibit the development of late blood vessel branches.^[15]^ Studies have found that Notch4 is expressed in tumor cells and is closely related to tumor invasion and metastasis and patient prognosis.^[16,17]^ Delta-like ligand 4 (DLL4) is found to be mainly expressed in the endothelial cells of the tumor vasculature and has an important role in the regulation of tumor angiogenesis.^[18,19]^ However, recent studies have reported extensive DLL4 expression observed in some tumor cells, and DLL4 is also associated with patient prognosis.^[20,21]^

The KAI1/CD82 protein is a member of TM4SF (transmembrane 4 superfamily) and is located on human chromosome 11p11.2.^[22]^ It was originally thought to be an inhibitory gene for metastasis of prostate cancer cells.^[22,23]^ It mediates between cells as well as signal transduction between cells and ECM to exert biological effects.^[24]^ Many studies have shown that reduced KAI1/CD82 expression may be a useful marker for many human tumors metastases, invasion, and prognostic factors.^[24–26]^

Overall, studies on the association between tumor metastasis and prognosis suggest that VM, Notch4, DLL4, and KAI1/CD82 affect cancer progression. However, associations among VM, Notch4, DLL4, and KAI1/CD82 in NSCLC have not been widely reported. In our study, we examined the hypothesis that these factors are mutual correlated, and are related to metastasis and prognosis in NSCLC.

## 2 Methods

### 2.1 Patients and tissue samples

We collected 189 patients (median age: 58.5 years; range: 28–79 years) from the First Affiliated Hospital of Bengbu Medical College, (China) from January 2011 to December 2012, and they were treated for NSCLC. Samples of corresponding adjacent non-tumor tissues from all 189 tumor patients were removed. Patients who had received chemotherapy or radiation before surgical radical surgery were excluded. All selected cases were obtained with the written consent of the patient. The study was conducted in accordance with the guidelines of the Helsinki Declaration and was approved by the Ethics Committee of the Bengbu Medical College (BBMUEC 201718). We collected patients with complete clinical pathology data and follow-up information (by phone, mail or email every 6 months). We calculated the patient's overall survival (OS) time, which were measured from the date of surgery of the oncology patients to his/her death date or December 2017 (mean OS: 41.4 months; range: 4–84 months). According to the 8th edition of the American Joint Committee on Cancer (AJCC), we assessed tumor-node-metastasis (TNM) stage of NSCLC. According to the World Health Organization (WHO) standards, we evaluated the grade of NSCLC. The relevant parameters of statistics were shown in Table 1.

**Table 1:**
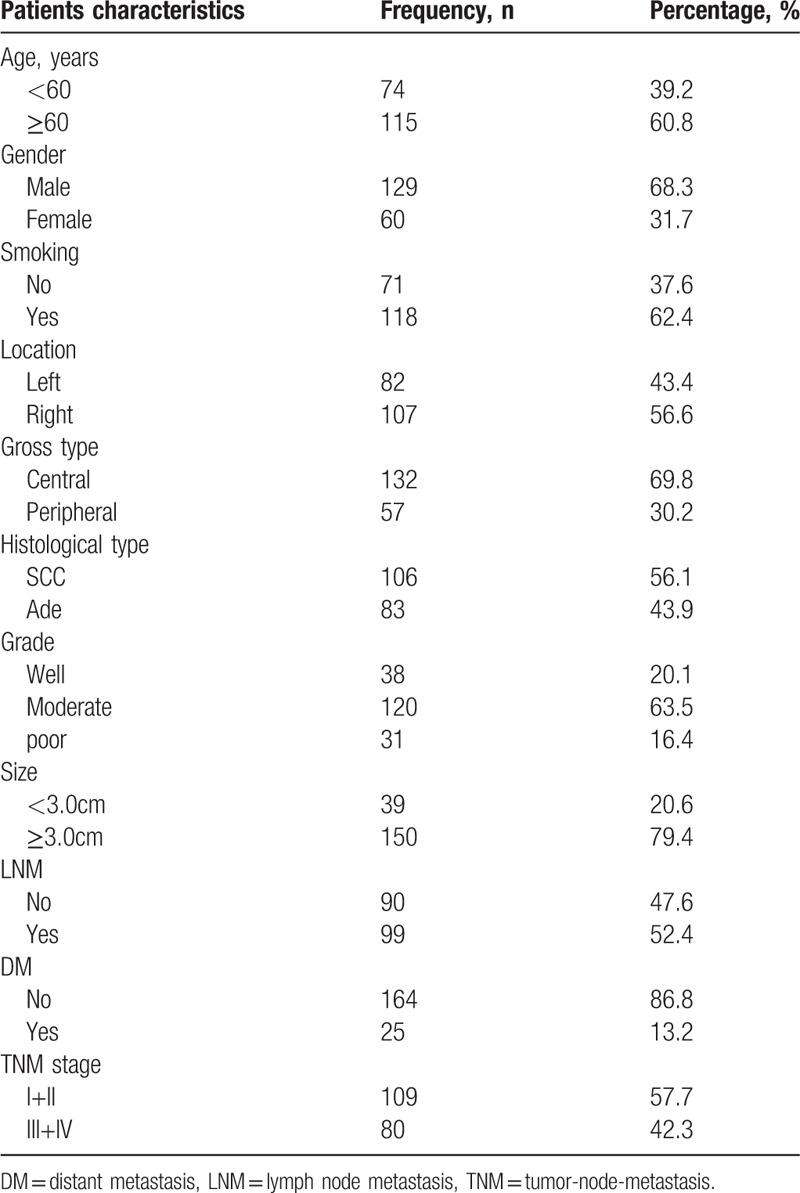
Patients characteristics.

### 2.2 Immunohistochemistry

We performed immunohistochemical staining of tumor tissues and corresponding non-tumor tissues according to the Elivision^TM^ Plus Test Kit Instructions (Lab Vision). We fixed all NSCLC and corresponding non-tumor tissue in 10% buffered formalin, in the next step, embedded them in paraffin. We cut the tissue block continuously into 4 μm thick sections. We deparaffinized all tissue sections and placed them in xylene solution and gradient ethanol for dehydration then washed the sections with phosphate buffered saline solution (PBS, pH 7.2) for 10 minutes. In order to block tissue endogenous peroxidase activity, we placed tissue sections in methanol containing 3% H_2_O_2_ for 10 minutes at room temperature. We placed the tissue sections in citrate buffer (pH 6.0) for antigen retrieval at a set temperature of 95°C for 30 minutes. After washing the tissue sections several times with PBS solution, all sections were quenched with goat serum for 30 minutes at room temperature. The sections were then incubated with mouse monoclonal antibodies against human CD34 (Abcam), Notch4 (Abcam), DLL4 (Abcam) and KAI1/CD82 (Abcam) at a set temperature of 37°C for 1 hour. We used Periodic Acid-Schiff (PAS)-CD34 dual staining to determine endothelial cells in glycosylated basement membranes of vessels, and we observed vessel-like (VM) structure in tumor and non-tumor tissues. We evaluated VM structure in the NSCLC tissues and the control non-tumor tissues according to Yue's method.^[27]^ Sections of all tissues were counterstained with hematoxylin, dehydrated, air dried, and finally fixed. KAI1/CD82 stains were mainly seen in cell membrane and cytoplasm. DLL4 and Notch4 stains were mainly seen in tumor cell cytoplasm.

### 2.3 Evaluation of staining

All sections staining results were evaluated semi-quantitatively method by 2 professional pathologists who were blind to patients’ clinical data and follow-up information. In order to reduce the effects of potential intratumoral heterogeneity of antibody expression, we selected 10 representative high-power-fields (HPF) from different regions of each NSCLC slide. The staining results were scored according to intensity staining (none staining, recorded as 0; weak staining, recorded as 1; moderate staining, recorded as 2; strong staining, recorded as 3) and extent (<11% positive cells mean, recorded as 1; 11%–50% positive cells mean, recorded as 2; 51%–75% positive cells mean, recorded as 3; >75% positive cells mean, recorded as 4). We obtained final scores by multiplying intensity and extent scores that ranged from 0 to 12. The final scores ≥3 was identified as positive staining result. For tissue sections that were positive staining results for all 4 of VM, Notch4, DLL4, and KAI1/CD82, we took an average value of the final score of each tissue section.

### 2.4 Statistical analysis

We analyzed the relationships between clinicopathological parameters and VM, Notch4, DLL4, and KAI1/CD82 using Fisher exact test or Chi-square test. Association between VM, Notch4, DLL4, or KAI1/CD82 was evaluated using Spearman correlate test. We analyzed effects of VM, Notch4, DLL4, or KAI1/CD82 on survival time using univariate and multivariate analyses. We analyzed independent prognostic factors by the multivariate Cox regression model. We used the Kaplan–Meier method with log-rank test to assess correlations between OS time and VM, Notch4, DLL4, or KAI1/CD82 results and clinicopathological characteristics. We performed all statistical analysis using SPSS 24.0 software for Windows (New York, IBM). A value of *P* <.05 was regarded as statistically significant.

## 3 Results

### 3.1 Association between VM, Notch4, DLL4, and KAI1/CD82 expression and clinicopathological characteristics

**Figure 1:**
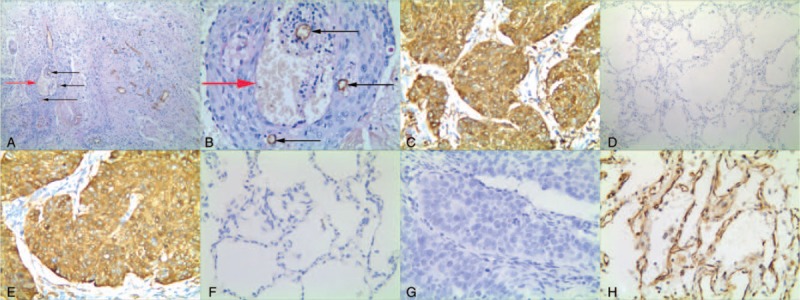
Positive staining of VM, or Notch4, or DLL4, or KAI1/CD82 in non-small cell lung cancer or the control tissue. (A) Positive staining of VM in the NSCLC tissue (100 magnification, red arrow is VM structure, black arrow is microvessel); (B) Positive staining of VM in the NSCLC tissue (400 magnification, red arrow is VM structure, black arrow is microvessel); (C) Positive staining of Notch4 in the cytoplasm of cancer cells (400 magnification); (D) Negative staining of Notch4 in the control tissue (100 magnification); (E) Positive staining of DLL4 in the cytoplasm of cancer cells (400 magnification); (F) Negative staining of DLL4 in the control tissue (400 magnification); (G) Negative staining of KAI1/CD82 in the NSCLC tissue (400 magnification); (H) Positive staining of KAI1/CD82 in the membrane and cytoplasm of the normal lung cells (400 magnification).

To assess the effects of VM, Notch4, DLL4, and KAI1/CD82 in NSCLC, the experimental results thereof were immunohistochemically detected for both NSCLC and corresponding normal lung tissue specimens. Moreover, we compared clinicopathological characteristics with these experimental data. The positive result rate of VM structure findings (small vessel structure, which is a lumen-like in NSCLC, the lumen-like structure was CD34-negative and PAS-positive staining result. The VM structure pattern included network, linear, and tubular, etc.) in the NSCLC specimens (37.0%, 70/189) was significantly higher than that in the corresponding normal lung tissues (0%, 0/189; *P* <.001; Fig. 1A and 1B). The positive rate of VM structure in NSCLC was positively related to tumor size, lymph node metastasis (LNM), DM, and TNM stage, but not patients’ age, gender, smoking, tumor location, gross type, histological type, or tumor grade (Table 2).

**Table 2:**
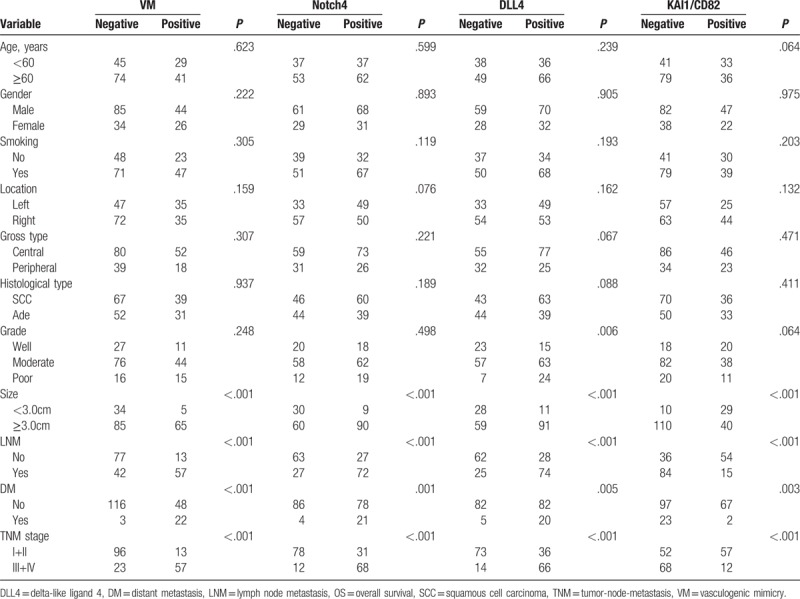
The correlation between VM, or Notch4, or DLL4, or KAI1/CD82 and clinicopathological characteristics in non-small cell lung cancer.

Similar to VM, Notch4+ expression was especially higher in NSCLC tissues (52.4%, 99/189) than that in the control normal lung tissues (6.9%, 13/189; *P* <.001; Fig. 1C and 1D). The positive rate of Notch4 expression in NSCLC was related to tumor size, LNM, DM, and TNM stage, but not patients’ age, gender, smoking, location, gross type, histological type, or tumor grade (Table 2).

The positive rate of DLL4 expression was higher in NSCLC tissues (54.0%, 102/189) than that in the control non-tumor tissues (7.9%, 15/189; *P* <.001; Fig. 1E and 1F). The positive rate of DLL4 expression was significantly associated with tumor grade, tumor size, LNM, DM, and TNM stage. No correlation was found between DLL4 expression and patients’ gender, age, smoking, location, gross type, or histological type (Table 2).

The positive expression rate of KAI1/CD82 expression was significantly lower in NSCLC tissues (36.5%, 69/189) than that in the control normal lung tissues (84.7%, 160/189; *P* <.001; Fig. 1G and 1H). The positive expression rate of KAI1/CD82 was inversely correlated with tumor size, LNM, TNM stage, and DM. No correlation was found between KAI1/CD82 positive expression and patients’ age, gender, smoking, location, gross type, histological type, or tumor grade (Table 2).

### 3.2 Univariate and multivariate analyses

**Figure 2:**
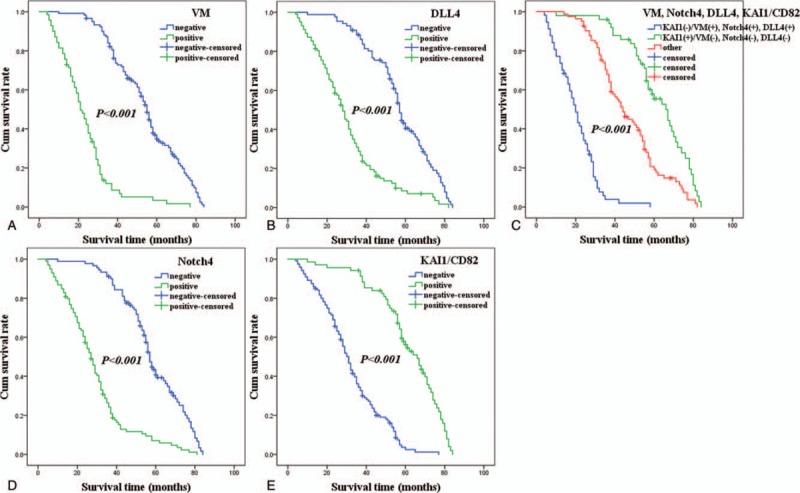
Kaplan–Meier analysis of the survival rate of patients with non-small cell lung cancer. The y-axis represents the percentage of patients; the x-axis, their survival in months. (A) Overall survival of all patients in relation to VM (log-rank = 126.642, *P* <.001); (B) Overall survival of all patients in relation to Notch4 expression (log-rank = 82.373, *P* <.001); (C) Overall survival of all patients in relation to DLL4 expression (log-rank = 62.322, *P* <.001); (D) Overall survival of all patients in relation to KAI1/CD82 expression (log-rank = 90.483, *P* <.001). In (A), (B), and (C) analyses, the green line represents patients with positive expression of VM, or Notch4, or DLL4 with a trend of worse survival time than the blue line representing the negative VM, or Notch4, or DLL4 group. In (D) analyses, the green line represents patients with positive expression of KAI1/CD82 with a trend of better survival time than the blue line representing the negative KAI1/CD82 group. (E) Overall survival of all patients in relation to the combination of KAI1/CD82, VM, Notch4, and DLL4 expression (log-rank = 177.119, *P* <.001). The blue line represents negative expression of KAI1/CD82 and positive expression of VM, Notch4, DLL4 and the green line represents positive expression of KAI1/CD82 and negative expression of VM, Notch4, DLL4. The red line represents other positive or negative expression of the proteins.

Follow-up data suggested that overall survival time (OST) was significantly lower in NSCLC patients with VM+ specimens (22.7±13.3 months) compared with VM- patients (52.3 ± 16.4 months; log-rank = 126.642, *P* <.001; Fig. 2A). Similarly, OST of Notch4+ patients (28.2 ± 16.3 months) was significantly lower than Notch4- patients (55.9 ± 15.2 months; log-rank = 82.373, *P* <.001; Fig. 2B). The OST of DLL4+ patients (29.4 ± 16.9 months) was significantly shorter than DLL4- patients (55.4 ± 16.1 months; log-rank = 62.322, *P* <.001; Fig. 2C). The OST of KAI1/CD82+ (59.5 ± 16.6 months) was significantly higher than those KAI1/CD82- specimens (30.9 ± 15.3 months; log-rank = 90.483, *P* <.001; Fig. 2D). The combination of KAI1/CD82− expression and VM+, Notch4+, and DLL4+ expression resulted in poorer prognoses than did the reverse combination (log-rank = 177.119, *P* <.001; Fig. 2E). In univariate analysis, OS time was significantly related to clinicopathological information, including tumor size (log-rank = 56.605, *P* <.001), LNM (log-rank = 109.789, *P* <.001), DM (log-rank = 104.058, *P* <.001), and TNM stage (log-rank = 142.119, *P* <.001) (Table 3).

**Table 3:**
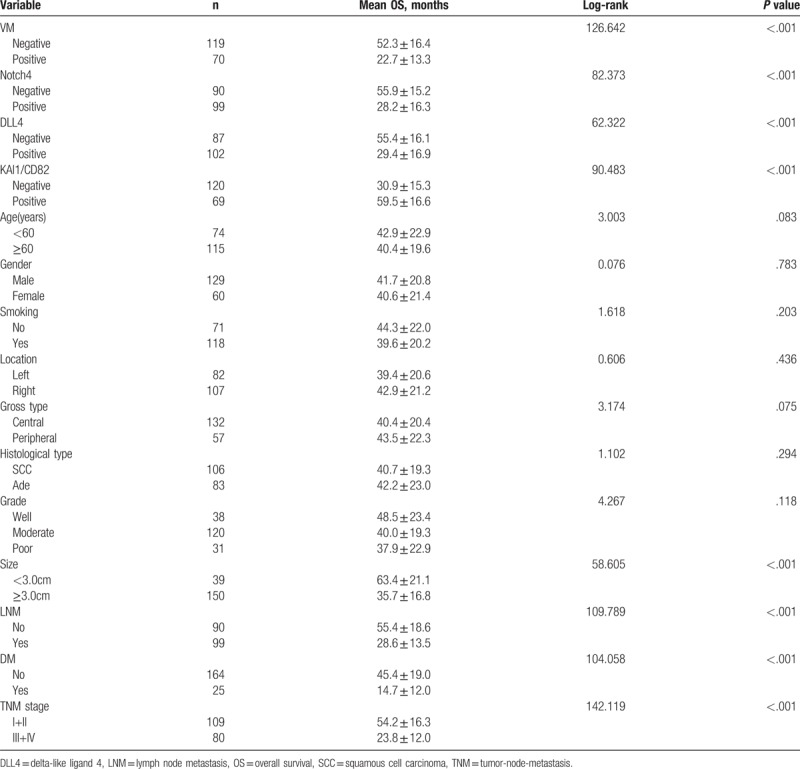
Results of univariate analyses of OS time.

Multivariate analysis demonstrated that VM+, Notch4+, DLL4+, and/or KAI1/CD82+ specimens, and tumor size, LNM, distant metastasis (DM), and TNM stage, were independent prognostic factors for NSCLC (Table 4).

**Table 4:**
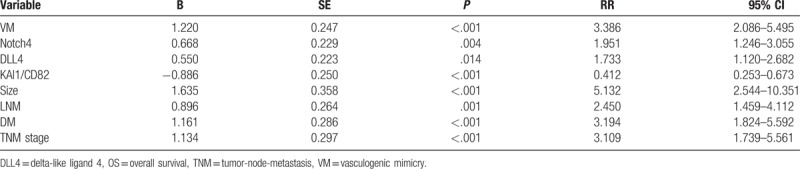
Results of multivariate analyses of OS time.

### 3.3 Correlation among VM, and expression of Notch4, DLL4, and KAI1/CD82 in NSCLC

Spearman correlation coefficient analysis indicated that negative correlations between KAI1/CD82+ expression and that of VM (*r* = −0.445, *P* <.001), Notch4 (*r* = −0.575, *P* <.001), or DLL4 (*r* = −0.579, *P* <.001). Expression of Notch4 was positively associated with a positive rate of VM (*r* = 0.622, *P* <.001) and DLL4 (*r* = 0.692, *P* <.001). The expression of VM and DLL4 showed a positive correlation (*r* = 0.489, *P* <.001) (Table 5).

**Table 5:**
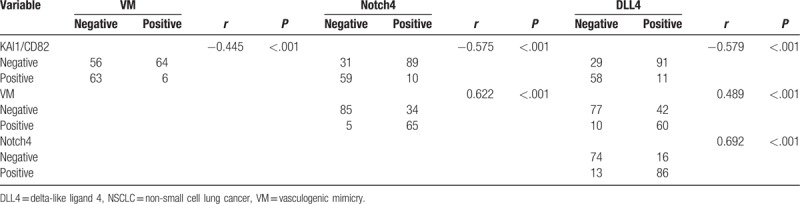
Correlation among VM, Notch4, DLL4, and KAI1/CD82 in NSCLC.

## 4 Discussion

Metastasis is a major cause of poor prognosis in NSCLC patients. The progression and metastasis of NSCLC are closely related to blood supply style and disorder of various molecules. In our study, we analyzed VM, Notch4, DLL4, and KAI1/CD82, to provide a new direction for investigating the metastasis and prognosis of NSCLC.

In this study, we found that VM was positively correlated with tumor size, LNM, DM, and TNM stage. Moreover, Kaplan–Meier survival analysis indicated that VM+ NSCLC patients had significantly lower OS time than did VM-patients. Our studies suggested that VM should play a vital role in NSCLC progression and metastasis, and it should also be considered as a very useful biomarker for clinical practice. Some studies indicated that VM may be one of the reasons for the failure of anti-angiogenesis therapy measure in clinical practice, and should be considered as a potential therapeutic target for NSCLC.^[28,29]^ Other researchers had also drawn similar findings.^[7–10]^

Notch4, a marker of Notch signaling pathway receptors, which is involved in the regulation of blood vessel formation and the remodeling and maturation of vascular networks.^[15]^ In this study, Notch4 expression was positively associated with tumor size, LNM, DM, and TNM stage. In addition, Kaplan–Meier survival analysis showed that Notch4+ NSCLC patients had significantly lower OST than did Notch4-patients. These results demonstrated that overexpression of Notch4 should promote NSCLC invasion, metastasis, and mean poor prognosis. Our findings are consistent with other researches, and including those of NSCLC and other cancers.^[13,16,17,30]^

DLL4 is one of the ligands that regulate the activities of Notch pathways.^[11,12]^ It is involved in the regulation of the formation and development of lymphatic vessels and blood vessels during embryonic development.^[31]^ DLL4 expression highly correlates with malignant tumor angiogenesis and metastasis.^[32]^ In this study, DLL4 expression was positively related to tumor grade, tumor size, LNM, DM, and TNM stage. Moreover, Kaplan–Meier survival curve demonstrated that DLL4+NSCLC patients had significantly lower OST than did DLL4- patients. The above results suggested that overexpression of DLL4 should play an important role in the process of invasion, metastasis, and prognosis of NSCLC. Our results are similar to other studies.^[20,21,32–34]^

KAI1/CD82 is thought to be a suppressor gene of tumor metastasis, which can inhibit tumor metastasis by mediating signal transduction between cells and between cells and ECM.^[24]^ Findings in this study also showed that KAI1/CD82 expression was significantly lower in NSCLC tissues than that in control tissues, and its expression was negatively correlated with tumor grade, LNM, DM, and TNM stage. Furthermore, Kaplan–Meier survival demonstrated that NSCLC patients with KAI1/CD82+ samples had significantly longer survival time than did KAI1/CD82- patients. These findings suggested that down-regulation of KAI1/CD82 should promote NSCLC progression and metastasis. Previous researches are similar to our experimental results.^[24–26,35–37]^

TNM stages of NSCLC provide therapeutic strategies for tumor patients but not provide exhaustive tumor biological behavior information about NSCLC. Therefore, it is vital to find effective and novel biomarkers to predict NSCLC biological behavior, metastasis, and patients’ prognosis. In our study, multivariate analysis showed that VM, expression of Notch4, DLL4, and KAI1/CD82, as well as TNM stages, tumor size, DM, and LNM, were independent prognostic factors for NSCLC patients (Table 4). Our findings thus demonstrated that VM, Notch4, DLL4, and KAI1/CD82 were reliable biomarkers for NSCLC, and especially in predicting tumor metastasis and prognosis.

NSCLC is the most common type of lung cancer. Tumor cells can induce angiogenesis when the tumor grows to a certain size. But when the angiogenic blood supply cannot meet the needs of rapid tumor growth, some tumor cells can mimic endothelial cells, and form VM. VM can provide nutrients and oxygen for invasive tumors to survive, and also provide new channel for tumor metastasis.^[6–10]^ The Notch signaling pathway plays an important role in the regulation of angiogenesis during embryonic development and adulthood. A growing body of research indicates that dysregulation of the Notch pathway promotes angiogenesis in tumors.^[11,15,18,19]^ Studies have shown that inhibition of Notch4 function down-regulates Nodal and VE-cadherin expression, and impairs VM network formation in invasive melanoma cells.^[38]^ high expression of Notch4 in A375 and MUM-2B melanoma cells can promote the formation of VM tubes.^[39]^ Moreover, Inhibition of Notch4 expression inhibits invasion and disrupts VM network formation by down-regulating Matrix metalloproteinases (MMPs) expression in hepatocellular carcinoma cells.^[40]^ Activation of the DLL4/Notch signaling pathway promotes MMPs expression and affects the progression of malignant tumors.^[41]^ Therefore, the Notch4/DLL4 signaling pathway can promote the formation of tumor VM structure, and further leads to the invasion and metastasis of malignant tumors. There is increasing evidence that cell surface adhesion proteins and ECM components are critical for tumor metastasis.^[42,43]^ KAI1/CD82 is cell membrane protein that bind to ECM or adhesion protein.^[24,44]^ Moreover, KAI1/CD82 can prevent angiogenesis by inhibiting the epithelial-mesenchymal transition (EMT), and further inhibit tumor invasion and metastasis.^[25]^

Overall, our findings indicate that complex interrelationships between the VM, Notch4, DLL4, and KAI1/CD82 in tumor progression. According to our current findings, we have reason to believe that these factors and their interrelationships are related to the metastasis and prognosis of NSCLC.

## 5 Conclusions

In summary, low expression of KAI1/CD82 combined with high expression of VM, Notch4, and DLL4 was found to be associated with metastasis and poor prognosis in NSCLC. Furthermore, combined detection of VM, Notch4, DLL4, and KAI1/CD82 are valuable factors of metastasis and prognosis in NSCLC.

## Author contributions

Yichao Wang, Shiwu Wu, Ruixue Yang, and Xiaolin Wang carried out the design, analysis of pathology and drafted the manuscript. Hongfei Ci, Lei Zhou, and Bo Zhu carried out sample collection and coordination. Danna Wang performed the immunohistochemical staining. All authors read and approved the manuscript.

**Conceptualization:** Yichao Wang, Shiwu Wu, Danna Wang.

**Funding acquisition:** Yichao Wang, Lei Zhou, Shiwu Wu.

**Investigation:** Yichao Wang, Shiwu Wu.

**Methodology:** Yichao Wang, Hongfei Ci, Lei Zhou, Bo Zhu, Shiwu Wu, Danna Wang.

**Project administration:** Yichao Wang, Lei Zhou, Shiwu Wu.

**Resources:** Yichao Wang, Shiwu Wu.

**Writing – original draft:** Yichao Wang, Shiwu Wu.

**Writing – review & editing:** Yichao Wang, Ruixue Yang, Xiaolin Wang, Shiwu Wu.
